# Improvement of post-thaw quality and fertilizing ability of bull spermatozoa using Rho kinase inhibitor in freezing extender

**DOI:** 10.3389/fvets.2023.1155048

**Published:** 2023-07-06

**Authors:** Mina Behnam, Reza Asadpour, Tohid Rezaei Topraggaleh, Hossein Hamali

**Affiliations:** ^1^Department of Clinical Science, Faculty of Veterinary Medicine, University of Tabriz, Tabriz, Iran; ^2^Reproductive Health Research Center, Clinical Research Institute, Urmia University of Medical Sciences, Urmia, Iran; ^3^Department of Anatomical Sciences, School of Medicine, Urmia University of Medical Sciences, Urmia, Iran

**Keywords:** ROCK inhibitor, semen quality, apoptosis, gene expression, embryo development

## Abstract

In this study, it was hypothesized that the addition of an appropriate concentration of Y-27632 (a ROCK inhibitor) to the freezing extender prevents cryopreservation-induced apoptosis and improves embryonic development after *in vitro* fertilization (IVF). Semen samples were collected from five fertile Simmental bulls using an artificial vagina twice a week for 4 weeks. Selected samples were pooled and diluted with Tris-egg-yolk-glycerol (TEYG) extender containing different concentrations of Y-27632 (0, 10, 20, 30, and 40 μM) and then frozen in liquid nitrogen. After thawing, computer-assisted semen analysis (CASA), plasma membrane integrity, and acrosome intactness were evaluated in terms of morphological abnormalities, intracellular generation of reactive oxygen species (ROS), DNA fragmentation, phosphatidylserine (PS) externalization, and apoptotic-related gene expression. Finally, groups of frozen and thawed spermatozoa were used for bovine oocyte IVF. The results show that the semen extender at a concentration of 20 μM Y-27632 effectively improved total motility (TM), curvilinear velocity (VCL), as well as the plasma membrane and acrosome integrity compared to the control group (*p* < 0.05). Intracellular ROS levels were significantly (*p* < 0.05) lower in samples treated with 30 μM Y-27632 compared to the control specimen. Furthermore, supplementation of the semen extender with 20 μM Y-27632 resulted in more viable spermatozoa compared with the control group (*p* < 0.05). According to qRT-PCR results, the expression levels of *BAX* and *CASPASE-9* genes in samples treated with 30 μM Y-27632 were significantly downregulated, while the expression of *BCL2* was increased compared to the control (*p* < 0.05). The results of IVF demonstrated that the treatment of frozen–thawed spermatozoa with 20 μM Y-27632 increased blastocyst rates compared to the control group (*p* < 0.05). In conclusion, the addition of 20 μM Y-27632 into the freezing extender can improve the functionality and the fertilizing capacity of frozen spermatozoa due to its antioxidative and anti-apoptotic properties.

## Introduction

The structure of the mammalian plasma membrane contains phospholipids, glycolipids, cholesterol, and intra-membrane proteins. These membranes play a protective role and exchange many substances between the intracellular and extracellular environments (1–3). Spermatozoa are comprised of multiple compartments enclosed within the acrosome, plasma membrane, and mitochondrial membranes, which act as physiological barriers that must remain intact to allow cell viability, especially after cryopreservation (4). However, the freezing/thawing process of spermatozoa makes them vulnerable to lipid peroxidation, crystal formation, and osmotic gradients, leading to irreversible damage to the plasma membrane of spermatozoa (5). On the other hand, the process of freezing and thawing leads to detrimental alterations in the physical and chemical structure of spermatozoa. Such unfavorable changes include damage to the ultrastructural organelles, making them more susceptible to morphological defects, DNA fragmentation (6), lipid peroxidation (7), development of abnormal acrosome (8), reduced ATP production, and impaired mitochondrial membrane potential (9). All of these factors eventually result in a decrease in the ability of these cells to fertilize (10).

According to previous studies, apoptosis-related markers are induced following cryopreservation in frozen spermatozoa (11, 12). Cryopreservation induces the externalization of phosphatidylserine (PS), membrane permeability, caspase activation, DNA fragmentation (13), as well as increased generation of ROS (14) in animal models, such as boars (15) and bulls (16). According to the literature, cryopreservation is highly associated with the activation of caspases, namely 3, 8, and 9 (17). Caspase-3 is an executioner caspase activated by initiator caspases when cleaved (12). This caspase-3 induces apoptosis by activating Rho-associated coiled-coil protein kinases (ROCK) of the serine–threonine kinase family (18, 19).

Rho A is a small Rho family GTPase protein that belongs to the Ras homolog gene family. Rho A has been implicated in a variety of cellular processes, including cell proliferation, death, migration, cell polarity, and cell–cell contact (20). Rho-associated protein kinase (ROCK) is an important downstream effector of Rho A. ROCKs have been found in non-neural tissues, such as testes, lung, and liver, as well as the head and tail of mammalian spermatozoa, whereas ROCK II is found in the brain, heart, and muscles (21, 22).

Recent studies have shown that Y-27632 is a selective and potent inhibitor of Rho-associated kinase (Roh/Rock) by controlling the expression of ROCK I and ROCK II (23). It has been shown that Y-27632 could improve embryonic stem cell survival rates (24) and enhance the recovery rate of embryonic stem cells following cryopreservation (24–26). In a study, the use of a ROCK inhibitor, Y-27632, in the feline semen extender improved sperm quality after post-thawing (27). The addition of 5 to 10 μM of Y-27632 to the semen extender of bulls improved post-thaw motility (28). Y27632 has been shown to protect cryopreserved hESCs from apoptosis, improve ESC survival, and promote colony formation in stem cells (29).

According to the above statements, RHO kinase is activated during cryopreservation and leads to the initiation of apoptosis in cells. We hypothesized that by using Y-27632, we could reduce apoptosis during the freezing and thawing process, mainly through pathways that regulate intrinsic and extrinsic apoptosis. Therefore, the primary goal of this study was to analyze the effects of different concentrations of ROCK inhibitor (Y-27632) on the quality of post-thawed spermatozoa and embryonic development following IVF with frozen–thawed spermatozoa, as well as the anti-apoptotic mechanism induced by ROCK inhibition.

## Materials and methods

### Animal

Semen was collected from five healthy and fertile Simmental bulls aged 3–4, which were kept at Nahadehaye Dami Jahed (NDJ Co., Karaj, Iran, 37 47′N, 50 55′E) under uniform feeding and management conditions. Forty ejaculates (eight samples per bull) were collected via an artificial vagina (AV, Neustadt/Aisch, Müller, Nuremberg, Germany) twice a week for 4 weeks. Semen volume, sperm concentration, and progressive motility percentage were recorded. To eliminate individual bull effects, semen volume ≥ 5 mL, sperm progressive motility ≥ 75%, abnormal sperm ≤ 10%, and sperm concentration ≤ 1 × 10^9^ were pooled and used in freezing experiments.

### Experimental design

Tris-egg-yolk-glycerol (TEYG) extender containing 2.42 g Tris-hydroxymethyl amino-methane, 1.36 g citric acid, 1.0 g fructose, 0.028 g penicillin, streptomycin (100 g/mL), 7% *v*/*v* glycerol, and 20% *v*/*v* egg yolk in 73 mL dd H_2_O (30) was prepared in a single batch and divided into 20 mL aliquots. The stock solution of Y-27632 (Cayman Chemical, Ann Arbor, MI; Cat. No. 717140) was previously prepared (10, 20, 30, and 40 μM in dimethyl sulfoxide (DMSO) and stored at − 20°C. Just before semen collection, Y-27632 was added to the TEYG extender in the volume required to achieve a final concentration of 0 μM (as a control group), 10, 20, 30, and 40 μM Y-27632. The range of dose levels was based on previous studies (27). Extended semen was loaded into 0.5 mL of French straw (IMV Technologies, L’Aigle, France) by using a filling and sealing machine (MPP Quattro, Minitube, Tiefenbach, Germany) with a final concentration of 25 × 10^6^ spermatozoa per straw, equilibrated for 4 h at 4°C and then frozen using a programmable freezer (Digital Cool, IMV Technologies, L’Aigle, France). Briefly, straws were cooled to − 10°C with freezing rates of − 5°C/min, from − 10 to − 100°C with freezing rates of − 40°C/min, and from − 100 to − 140°C with freezing rates of − 20°C/min. Straws were subsequently placed in liquid nitrogen (− 196°C) and stored for at least 4 weeks. Straws were thawed in a 37°C water bath for 40 s and kept at 37°C for 5 min until evaluation.

### Computer-assisted semen analysis

Sperm kinematic parameters were analyzed using a computer assisted sperm analyzer (CASA, AndroVision^®^, minitube). The CASA was set for bull spermatozoa to take 30 images at a rate of 60 frames per second (60 Hz), with settings for a minimum cell size of 6 μm^2^ and a minimum head brightness of 180 intensities. The percent of progressive motility (PM, %) was recorded for each sample. Sperm were deemed motile when their average path velocity (VAP) was ≥ 20 μM/s and progressively motile when their straightness index (STR) was ≥ 80% (31). From each sample, an aliquot was diluted (1:10) with prewarmed Tris buffer, and then a droplet (3 μL) of the semen sample was put on a pre-warmed Leja 4-analysis chamber slide with a depth of 20.0 μm (Leja, Nieuw-Vennep, Netherlands). Curvilinear velocity (VCL, μM/s), straightness (STR = VSL/VAP, %), average path velocity (VAP, μM), linearity (LIN = VSL/VCL, %), straight-line velocity (VSL, μM/s), beat cross-frequency (BCF, Hz), amplitude of lateral head displacement range (ALH, μM), and wobble (WOB = VAP/VCL, %) were measured in at least 1,000 spermatozoa in five random fields (32).

### Assessment of cell membrane integrity and morphology

The hypoosmotic swelling test (HOST) was performed to assess the plasma membrane function of spermatozoa (33). The evaluation was performed by incubating an aliquot (10 μL) of the sperm sample with 100 μL of a 100 mOsm/L hypoosmotic solution (1.9 mM sodium citrate and 5 mM fructose dissolved in 100 mL distilled water) at 37°C for 30 min. The solution was then loaded onto a pre-warmed glass slide with a coverslip in the amount of 15 μL. Two hundred sperm in each sample were calculated under a phase contrast microscope (AxioScope A1, Zeiss, Oberkochen, Germany) to assess swelling and curved tails.

Hancock’s solution was used to evaluate the morphology of frozen–thawed spermatozoa. For this test, 10 μL of an aliquot of semen was mixed with 1,000 μL of Hancock’s solution (150 mL sodium saline solution, 150 mL buffer solution, 62.5 mL formalin (37%), and 500 mL double-distilled water). Briefly, 10 μL of this mixture was placed on a glass slide and covered with a coverslip. A total of 200 sperm were counted in each smear at a magnification of 100 × (oil, immersion) using a phase contrast microscope (AxioScope A1, Zeiss, Oberkochen, Germany). Three replicates were done for each sample, and the percentage of sperm with abnormal morphology such as head abnormalities, midpiece abnormalities, tail abnormalities, and cytoplasmic droplets was calculated.

### Evaluation of acrosome integrity

To perform an acrosome integrity test, 10 μL of sperm were prepared at a concentration of 3–5 × 10^6^ cells/mL of semen into a 1.5 mL microtube. The semen samples were dissolved in 100 μL of ethanol (96%) and held at room temperature for 60 min. The sperm suspension (10 μL) was kept on a glass slide to allow ethanol to evaporate. Then, 30 μL of fluorescein-conjugated *Pisum sativum* agglutinin (FITC-PSA, 50 μL/mL) was applied on the slide so that the entire surface of the slide was coated with the solution. Then the slide was incubated at 4°C for 1 h, and at the end, it was washed 10 times with distilled water. Thereafter, the slide was dried, a drop of glycerol was poured onto the slide, and 200 sperm were counted per slide by a fluorescence microscope (BX51, Olympus, Tokyo, Japan) at 400 × magnification. The cell population examined under the microscope was divided into two categories: sperm that exhibit green fluorescent in the head area are considered to be intact acrosomes, whereas those that do not show green fluorescent in the head area or a green fluorescent band at the equatorial level are considered to be damaged or disrupted acrosomes (34). At least three replicates were included in each group.

### Sperm DNA fragmentation

Sperm DNA integrity has been determined using a Sperm DNA Fragmentation Assay Kit (SDFA; ACECR, Tehran, Iran) according to the manufacturer’s instructions. Low-melting-agarose (LMA) was placed inside the microtube for 5 min at a temperature of 90–100°C to become a gel, then 30 μL of each sperm sample was combined with 70 μL of LMA, and then the resulting mixture was placed on slides. It was specially placed, covered with a coverslip, placed in a refrigerator (4°C) for 5 min, and processed with a denaturation solution and a lysis solution for 7 and 15 min, respectively. Slides were dehydrated by increasing the concentration of ethanol (70, 90, and 100%) for 2 min for each concentration. After drying, slides were sequentially stained with kit solutions. At least 200 sperms were examined with a light microscope (Labomed, United States) at 100x objective magnification, and sperms with a large or medium halo were classified as intact chromatin, and sperms without a halo or small halo were classified as fragmented sperm (35).

### Detection of lipid peroxidation

The concentration of malondialdehyde (MDA) as an indicator of lipid peroxidation (LPO) was measured in seminal plasma using the thiobarbituric acid (TBA) reaction (36) with malondialdehyde using an MDA assay kit (Tali Gene Pars, Isfahan, Iran) according to the manufacturer’s instructions. After equilibration to room temperature, reagents were added to the samples. Briefly, 1 mL of diluted semen (25 × 10^6^ sperm/mL; three straws per replicate) was mixed with 1 mL of cold 20% (*w*/*v*) trichloroacetic acid for protein precipitation. The precipitate was pelleted by centrifugation (4,000 rpm for 15 min) and 750 μL of the supernatant was incubated with 750 μL of 0.67% (*w*/*v*) TBA in a boiling water bath at 95°C for 10 min. After cooling, the absorbance was evaluated at 532 nm with a spectrophotometer (T80 UV/VIS PG Instruments Ltd., United Kingdom), and the MDA level was reported as nmol/mL of seminal plasma.

### Flow cytometric analysis

A FACSCalibur (Becton Dickinson Biosciences, San Jose, CA, USA) Flow cytometric set was used to evaluate apoptotic-like changes and intracellular ROS production with an air-cooled argon laser operated at 488 nm excitation and 15 mW (37). A minimum 10,000 spermatozoa were counted for each assay at a flow rate of 100 cells/s. The sperm population was gated using 90° and forward-angle light scatter to exclude debris and aggregates. For apoptosis assay, FL1 with a 530/30 nm filter was utilized to detect green fluorescent (GF), and FL2 with a 582/42 nm filter was utilized to recognize red fluorescent (RF). For evaluation intracellular ROS production red fluorescence were detected with FL2 detector (525–625 nm). The analysis of flow cytometry data was performed using FlowJO software (Treestar, Inc., San Carlos, CA, United States).

### Apoptosis-like changes

To determine the index of apoptotic changes in sperm, the Annexin-V and PI kit (IQP, Groningen, Netherlands) was used to detect phosphatidylserine (PS) externalization according to the manufacturer’s instructions. Briefly, the samples were first washed with calcium buffer, and then 10 μL of Annexin-V conjugated to fluorescein isothiocyanate (Annexin-V FITC 0.01 mg/mL) was added to 100 μL of sperm sample and incubated on ice for 20 min in a dark room. Then 10 μL of propidium iodide (PI) was added to the sperm suspension and incubated on ice for 10 min. Then, the suspension was evaluated using a flow cytometer. According to flow cytometric methods, the sperm subpopulation was divided into four groups: I: viable, non-apoptotic cells negative for both Annexin-V and PI; II: early-apoptotic cells positive for Annexin-V but negative for PI; III: late-apoptotic cells positive for Annexin-V and PI; IV: necrotic cells negative for Annexin-V and positive for PI (38).

### Evaluation of ROS production

The intracellular ROS was assessed using a dihydroethidium (DHE; D 7008, Sigma-Aldrich, United States, St Louis, MO, United States). DHE is oxidized by the free intracellular O_2_^–•^ into ethidium bromide that binds to the DNA and emits red fluorescence. To detect intracellular O_2_-, semen samples (1–2 × 106 sperm/mL) were incubated with 1.25 μM of DHE in the dark for 20 min at room temperature (25°C). Each sample was analyzed using flow cytometry (Becton Dickinson, FACScan, San Jose, CA, United States) with a 488 nm argon laser. Data were expressed as the percentage of fluorescent spermatozoa (39).

### Gene expression analysis

Twenty-five semen straws (five straws per group) have been thawed in a water bath at 37°C for 30 s. Thereafter, the semen samples were washed three times with saline (PBS), and the pellets were obtained by centrifuging at 1,600 rpm for 5 min. Quantitative reverse transcription PCR (RT-qPCR) was accomplished to detect the change in the expression of apoptosis-related genes (*CASPASE-3*, *CASPASE-8*, *CASPASE-9*, *BAX, BCL2*, and *ROCK I*) using the primer sequence listed in Table 1. Briefly, total RNA was extracted using TRIzol reagent (Anacell, Iran) and was subsequently treated with DNase (Sinaclon Bioscience) to avoid amplification of contaminated genomic DNA. The RNA pellet was dissolved in RNase-free water (50 μL) and its concentration and purity were assessed by OD at 260 and 260/280 nm using a NanoDrop 2000 spectrophotometer (Thermo Scientific, respectively). A total of 1–2.5 ng of RNA were used for complementary DNA (cDNA) synthesis according to the protocols of the manufacturer’s cDNA synthesis kit (ANACELL, Iran). Reaction conditions followed the manufacturer’s instructions: 70°C for 5 min, 42°C for 60 min, followed by inactivation of the enzyme at 70°C for 10 min. After the reaction, cDNA samples were diluted with nuclease-free water, and stored at − 20°C. A total of 20 μL of cDNA gained from this reaction was used for RT-qPCR. RT-qPCR was carried out using cDNA samples, specific primers, and SYBER Green by Rotor-Qiagen. A 15-min denaturation step at 95°C was followed by 40 PCR cycles (95°C for 15 s, 65°C for 20 s, 76°C for 20 s, and 75°C for 14 s). All qPCR reactions were accomplished in duplicate with a non-template control (NTC) to check for genomic DNA (gDNA) contamination. RT negative controls were performed to check for DNA contamination in cDNA synthesis. Samples were run in triplicate, and the relative gene expression was calculated using the comparative threshold cycle (Ct) and normalized to expression of GAPDH (**2**^-∆∆CT^) as a housekeeping gene. Results are expressed as fold change relative to the control.

**Table 1:** Primes used for real time-PCR.

Gene name	Direction	Sequence (5′–3′)	TM	Amplicon (bp)	Accession number
BCL 2	F	CTTTGAGTTCGGAGGGGTCAT	59.72	158	NM_001166486.1
BCL2	R	ATACAGCTCCACAAAGGCGTC	60.68	158	
BAX	F	CCCTTGGCTGAGTCGCTGA	61.59	72	XM_015458140.2
BAX	R	GGTGTCCCAAAGTAGGAGAGG	59.44	72	
CASPASE 3	F	TCGTAGCTGAACCGTGAGTGA	61.15	151	XM_010820245.3
CASPASE 3	R	TGTCTCCTCCTCGGGGTAGC	62.57	151	
CASPASE 8	F	GCGTTTCCAACCCCACTTC	59.34	167	XM_005202615.4
CASPASE 8	R	ACACAGTCCTGGCAACATCC	60.25	167	
CASPASE 9	F	GATCAGGCCAGGCAGCTAAT	59.89	156	NM_001205504.2
CASPASE 9	R	CGGCTTTGATGGGTCATCCT	60.11	156	
ROCK I	F	TGAACAGGAAGGCGGACATA	58.73	166	XM_002697789.6
ROCK I	R	GCGAGGATAGACGAGAGTCG	59.49	166	
GAPDH	F	GGTCACCAGGGCTGCTTTTA	60.25	222	NM_001034034.2
GAPDH	R	CCAGCATCACCCCACTTGAT	60.03	222	

F, Forward primer; R, reverse primer. BCL2: B-cell lymphoma 2; BAX: BCl-2 Associated X-protein; ROCK I: Rho-associated, coiled-coil-containing protein kinase I; GAPDH: GAPDH glyceraldehyde-3-phosphate dehydrogenase.

### *In vitro* embryo production (IVEP)

The procedures for ovary collection, transportation, and IVM have been recently described in detail (40). Briefly, bovine ovaries were brought to the laboratory from a local slaughterhouse, suspended in Dulbecco’s phosphate-buffered saline (DPBS) at 35–37°C for 2 h, and then the ovaries were washed with normal saline at 36°C. COCs were aspirated from follicles with a diameter of 2–8 mm using an 18-gauge needle and a sterile 10 mL disposable syringe. The 502 bovine COCs that were used in this study had three layers of unexpanded cumulus and three layers of uniform ooplasm, which were washed three times in washing medium. A group of 20 COCs per well were transferred to four-well dishes and matured in IVM medium (TCM-199 supplemented with 22 μg/mL pyruvate, 10% (*v*/*v*) FBS, 50 μg/mL HCG, 0.5 μg/mL FSH, 1 μg/mL estradiol, and 0.05 μg/mL gentamicin) for 22 h at 38.5°C, 5% CO_2_ in a humidified atmosphere. Motile spermatozoa were obtained by the swim-up procedure previously described Parrish et al. (41). After thawed semen samples at 37°C within 30 s, 1 mL of semen samples was layered under 1 mL aliquots of Sperm-TALP (Tyrode’s albumin lactate pyruvate) medium in a 15 mL falcon conical tube. After 1 h incubation at 39°C, the top 0.85 mL of medium was removed and diluted to a final volume of 5 mL with Sperm-TALP medium, centrifuged at 120 *g* for 10 min and the supernatant discarded. The concentration of the sperm pellet was then determined and sample re-suspended in 100 μL of IVF-TALP medium and was added to each fertilization drop, giving a total concentration of 2 × 10^6^ sperm/mL. Ten COCs were co-incubated with sperm in a drop of 50 μL IVF medium (Tyrode’s albumin-lactate-pyruvate supplemented with 6 mg/mL BSA, 2 mM penicillamine, 1 mM hypotaurine, 0.25 mM epinephrine, and 10 mg/mL heparin) in four-well plates under mineral oil for 24 h at 38.5°C, 5% CO2, in a humidified atmosphere (42). After 24 h, putative zygotes were placed in vortex chambers to remove excess spermatozoa and zona-adherent cumulus cells. Embryos (*n* = 10 per droplet) were then cultured in 50 μL drops of IVC medium (Synthetic oviductal fluid (SOF) medium supplemented with BME essential amino acid and MEM non-essential amino acids, citrate, and inositol) and fatty acid free BSA (3 mg/mL) (43). The drops were covered with mineral oil and incubated at 38.5°C with 5% CO2 and 5% O2, 90% N2 in a humidified atmosphere, and the culture medium was refreshed every 48 h. Embryo development was assessed using a stereomicroscope (Luxeo 4D Stereozoom Microscope, Labomed, CA, United States) at the zygote, cleavage, morula, and blastocyst stages at days 1, 2, 5, and 7 or 8 post-inseminations, respectively. On day 2 after the initiation of fertilization, the cleavage rate was assessed by the division rate percentage of cleaved embryos/presumed zygotes, at day 5 after fertilization, the morula rate was calculated by the division rate percentage of morula embryos/ presumed zygotes; and the blastocyst rate was assessed by the division rate percentage of blastocysts/presumed zygotes on days 7–8 after insemination. The days of fertilization were considered Day 0 (44).

### Statistical analysis

SPSS (IBM SPSS Statistics for Windows, V.25.0, Armonk, NY: IBM) was used for data analysis, and Graph-Pad Prism software v.8 (San Diego, United States) was used for graph drawing. Data were checked for normality with the Shapiro-Wilks test and then analyzed using parametric and non-parametric statistical methods. The results obtained with the Levene’s test indicated that the variances were homogeneous. Statistical differences among the groups were analyzed by one-way analysis of variance (ANOVA), followed by Tukey’s *post hoc* test for comparison between groups. Using the Chi-square test, the percentage of embryonic development, including the percentage of cleavage, morula, and blastocyst, was compared between groups. The significance level was set at *p* < 0.05. All data are shown as mean ± SEM.

## Results

### Motion characteristics

The effect of Y-27632 concentrations on the motility of thawed sperm is shown in Table 2. The addition of 20 μM Y-27632 to the freezing extender resulted in a significantly higher total motility (TM) than the other treatment groups (*p* < 0.05). The addition of different concentrations of Y-27632 to the freezing extender did not affect the progressive motility (PM) of post-thaw spermatozoa compared to the control group (*p* > 0.05). Y-27632 at a concentration of 20 μM in freezing extender significantly (*p* < 0.05) increased the VCL in post-thaw spermatozoa compared to the control and cells treated with 40 μM Y-27632. The addition of 30 μM Y-27632 to the freezing extender significantly increased (*p* < 0.05) the straight-line velocity (VSL) of post-thaw spermatozoa compared to the control group and cells treated with 40 μM Y-27632; however, no significant difference was observed between spermatozoa treated with 20 and 30 μM Y-27632. A statistically significant (*p* < 0.05) increase was found in the average path velocity (VAP) of spermatozoa treated with 20 μM Y-27632 compared to those treated with 10 and 40 μM Y-27632, or the control group. The highest value for beat cross frequency (BCF) was observed in post-thaw sperm after adding 20 μM Y-27632 compared to the control group and cells treated with 40 μM Y-27632 (*p* < 0.05). The value of the amplitude of lateral head displacement (ALH) was significantly higher (*p* < 0.05) in spermatozoa treated with 30 μM Y-27632 groups compared to the control group and cells treated with 40 μM Y-27632, while no significant difference was detected between spermatozoa treated with 30 μM and those treated with 10 and 20 μM Y-27632. Progressive motility (PM), linearity (LIN), straightness (STR) and wobble (WOB) were not affected with different concentrations of Y-27632 (*p* > 0.05).

**Table 2:** Means ± standard error of the mean (SEM) of motion characteristics assessed by CASA in frozen–thawed sperm after ROCK inhibitor (Y-27632) treatments.

Concentration of Y-27632						
Variable (unit)	0 μM (Control)	10 μM	20 μM	30 μM	40 μM	*p*-Value
TM (%)	47.70 ± 1.41^ab^	47.16 ± 0.57^a^	54.28 ± 1.51^b^	47.14 ± 0.81^a^	41.69 ± 2.44^a^	*p* = 0.003
PM (%)	30.07 ± 0.18	32.53 ± 0.25	33.52 ± 0.54	34.11 ± 1.71	31.55 ± 0.74	*p* = 0.056
VCL (μm/s)	99.28 ± 0.92^ab^	102.12 ± 1.57^abc^	106.99 ± 1.50^c^	105.71 ± 2.28^bc^	97.51 ± 1.20^a^	*p* = 0.007
VSL (μm/s)	43.38 ± 0.29^a^	44.23 ± 0.53^ab^	48.88 ± 1.88^bc^	49.54 ± 1.14^c^	43.85 ± 0.37^a^	*p* = 0.003
VAP (μm/s)	52.72 ± 0.17^b^	53.22 ± 0.96^b^	58.08 ± 1.41^a^	56.54 ± 2.27^ab^	52.88 ± 0.28^b^	*p* = 0.042
BCF (Hz)	11.89 ± 0.14^ab^	12.94 ± 0.22^abc^	14.07 ± 0.63^c^	13.75 ± 0.67^bc^	11.65 ± 0.17^a^	*p* = 0.009
ALH (μm)	1.25 ± 0.01^ab^	1.33 ± 0.02^abc^	1.35 ± 0.02^bc^	1.37 ± 0.01^c^	1.24 ± 0.03^a^	*p* = 0.008
LIN (%)	44.00 ± 0.00	43.33 ± 0.66	45.66 ± 1.33	45.33 ± 1.20	45.00 ± 0.57	*p* = 0.37
STR (%)	82.33 ± 0.66	83.00 ± 0.57	84.33 ± 1.20	84.33 ± 0.88	83.33 ± 0.33	*p* = 0.36
WOB (%)	53.33 ± 0.33	52.33 ± 0.33	54.33 ± 0.66	53.00 ± 1.00	54.00 ± 0.57	*p* = 0.25

TM: total motility; PM: progressive motility; VCL: curvilinear velocity; VSL: straight line velocity; VAP: average path velocity; BCF: beat cross frequency; ALH: amplitude lateral head displacement, LIN: linearity; STR: straightness. WOB: wobble. The values are shown as means ± Standard Error of the Mean (SEM). ^a,b,c^ different superscripts in the same row indicate differences between treatment groups (*p* < 0.05); each experiment was performed at least three times and underwent statistical analysis. –, no significant difference (*p* > 0.05).

### Morphology

The morphological parameters of frozen–thawed spermatozoa are presented in Table 3. After thawing, the extender treated with 10 μM Y-27632 had a significantly (*p* < 0.05) higher proportion of normal sperm than those treated with 40 μM Y-27632. The results also indicated that the addition of different concentrations of Y-27632 had no significant effect on the percentage of mid-piece, head, and tail defects as well as proximal cytoplasmic droplets of post-thaw spermatozoa compared to the control group (*p* > 0.05).

**Table 3:** Effect of different concentrations of ROCK inhibitor (Y-27632) on morphological abnormalities in frozen–thawed spermatozoa of Simmental bulls.

Concentration of Y-27632						
Variable (unit)	0 μM (Control)	10 μM	20 μM	30 μM	40 μM	p-value
Normal morphology (%)	84.00 ± 2.08^a^	89.33 ± 0.33^b^	89.00 ± 1.00^ab^	85.66 ± 1.33^ab^	82.66 ± 2.02^a^	*p* = 0.037
Head defect (%)	4.00 ± 1.52	2.66 ± 0.66	3.33 ± 0.88	2.66 ± 0.88	5.66 ± 0.66	*p* = 0.242
Abnormal midpieces (%)	2.33 ± 0.88	1.00 ± 0.57	1.00 ± 0.57	2.33 ± 0.88	2.00 ± 0.57	*p* = 0.492
Abnormal tails (%)	9.00 ± 2.64	7.00 ± 0.57	6.00 ± 2.08	8.33 ± 0.88	8.33 ± 0.33	*p* = 0.681
Proximal cytoplasmic droplet (%)	0.66 ± 0.33	00.00 ± 0.00	0.66 ± 0.33	1.00 ± 0.57	1.33 ± 1.33	*p* = 0.720

The values are shown as means ± standard error of the mean (SEM). ^a,b,c^Different superscripts in the same row indicate differences between treatment groups (*p* < 0.05); each experiment was performed at least three times and underwent statistical analysis. –, no significant difference (*p* > 0.05).

### Viability, plasma membrane, acrosomal and DNA integrity

Table 4 shows the average viability, plasma membrane integrity (PMI), acrosome integrity, and sperm DNA fragmentation following the treatment with different concentrations of Y-27632. When compared to spermatozoa treated with 40 μM Y-27632 and the control group, cells treated with 20 μM Y-27632 substantially increased (*p* < 0.05) post-thaw spermatozoa viability and PMI. The addition of 20 and 30 μM Y-27632 to the freezing extender significantly (*p* < 0.05) increased the percentage of acrosome integrity compared to spermatozoa treated with 40 μM Y-27632 and the control group. Furthermore, a decrease in the percentage of DNA fragmentation was observed after supplementation of the freezing extender with 20 μM Y-27632; however, no significant difference was found in the percentage of DNA fragmentation between different concentrations of Y-27632 and the control group (*p* > 0.05).

**Table 4:** Effect of different concentrations of Y-27632 on viability, plasma membrane integrity, acrosome integrity, sperm DNA integrity of frozen thawed sperm.

Concentration of Y-27632						
Variable (unit)	0 μM (Control)	10 μM	20 μM	30 μM	40 μM	*p*-Value
Viability (%)	60.00 ± 1.00^a^	64.66 ± 0.0.33^ab^	68.33 ± 1.66^b^	64.66 ± 2.33^ab^	60.66 ± 1.76^a^	*p* = 0.022
PMI (%)	59.66 ± 1.33^a^	64.00 ± 2.08^ab^	70.66 ± 1.76^b^	65.33 ± 1.20^ab^	61.66 ± 1.20^a^	*p* = 0.005
AI (%)	77.66 ± 1.33^ab^	84.00 ± 2.08^bc^	89.00 ± 0.57^c^	85.33 ± 1.20^c^	76.66 ± 1.20^a^	*p* = 0.0003
DNA fragmentation (%)	9.22 ± 1.80	9.01 ± 1.15	4.41 ± 1.08	6.02 ± 1.98	7.20 ± 0.70	*p* = 0.158

Values are presented as the means ± Standard Error of the Mean (SEM). ^a,b,c^Different superscripts in the same row indicate differences between treatment groups (*p* < 0.05); each experiment was performed at least three times and underwent statistical analysis. –, no significant difference (*p* > 0.05). PMI: Plasma membrane integrity; AI: Acrosome integrity and DNA fragmentation.

### Production of ROS and MDA formation

**Figure 1:**
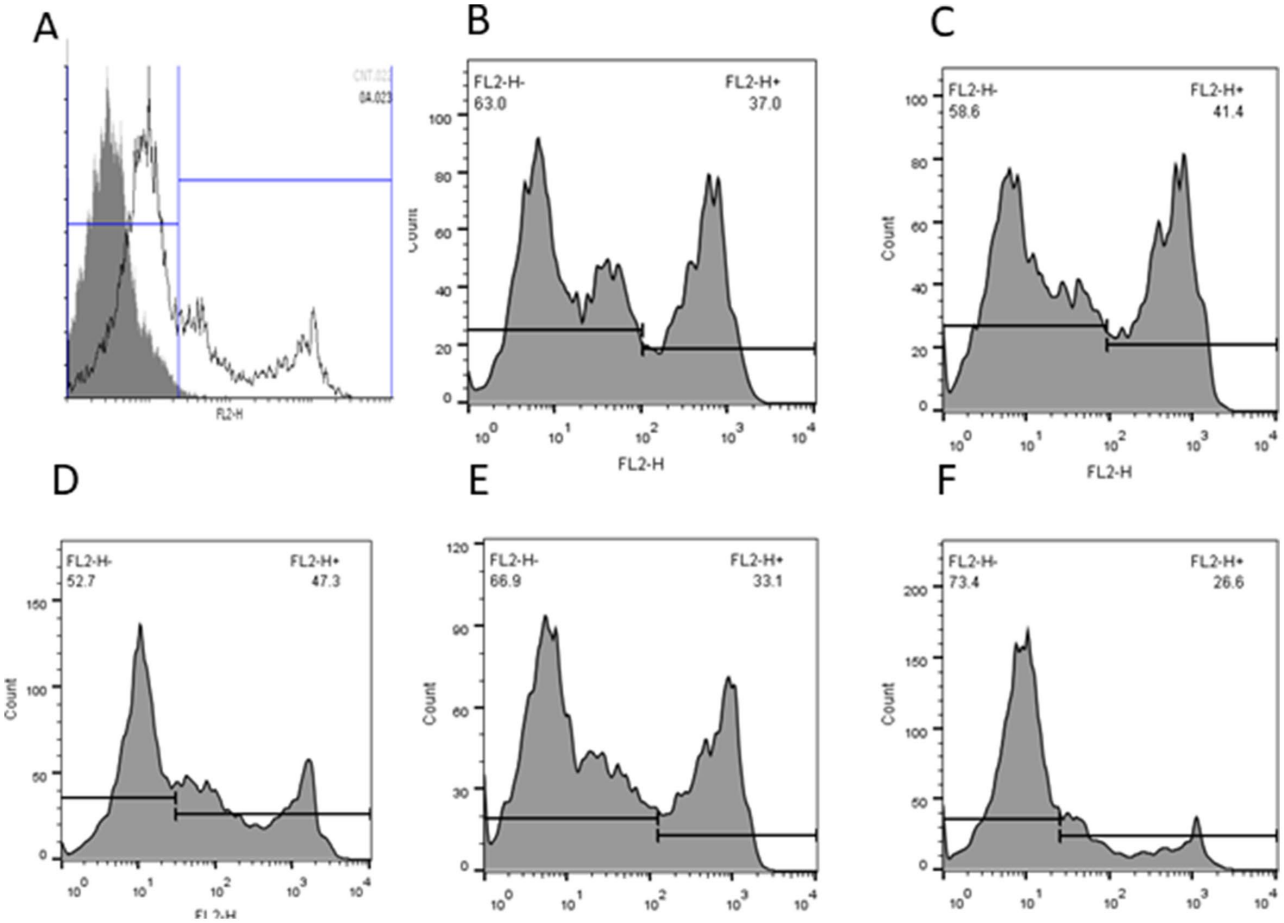
The intracellular reactive oxygen species (ROS) of frozen–thawed spermatozoa with or without Y-27632 (**A**: negative control; **B**: control; **C**: 10 μM, **D**: 20 μM; **E**: 30 μM, and **F**: 40 μM Y-27632) was detected by flow cytometry. Data were expressed as the percentage of fluorescence emitted by stained spermatozoa.

Table 5 shows the percentage of intracellular ROS production and MDA levels after treatment with different concentrations of Y-27632. The level of intracellular ROS was significantly (*p* < 0.05) decreased in cells treated with 30 μM Y-27632 compared with the control group (Table 5; Figures 1A–E). MDA levels were significantly reduced (*p* < 0.05) in post-thaw spermatozoa treated with the freezing extender containing 20 μM Y-27632 compared to cells treated with 30 and 40 μM Y-27632 and the control group.

**Table 5:** Effect of different concentrations of Y-27632 on, Reactive oxygen species production and malondialdehyde (MDA) level of frozen thawed sperm.

Concentration of Y-27632						
Variable (unit)	0 μM (Control)	10 μM	20 μM	30 μM	40 μM	*p*-Value
ROS (%)	44.70 ± 3.91^b^	39.96 ± 2.55^ab^	33.13 ± 2.44^ab^	27.43 ± 4.03^a^	33.23 ± 3.06^ab^	*p* = 0.030
MDA (nmol/mL)	3.96 ± 0.12^c^	2.53 ± 0.06^ab^	2.16 ± 0.06^a^	2.60 ± 0.05^b^	3.76 ± 0.12^c^	*p* = 0.001

Values are presented as the means ± standard error of the mean (SEM). ^a,b,c^Different superscripts in the same row indicate differences between treatment groups (*p* < 0.05); each experiment was performed at least three times and underwent statistical analysis. –, no significant difference (*p* > 0.05). ROS: reactive oxygen species; MDA: malondialdehyde (MDA) production.

### Apoptosis-like changes

**Figure 2:**
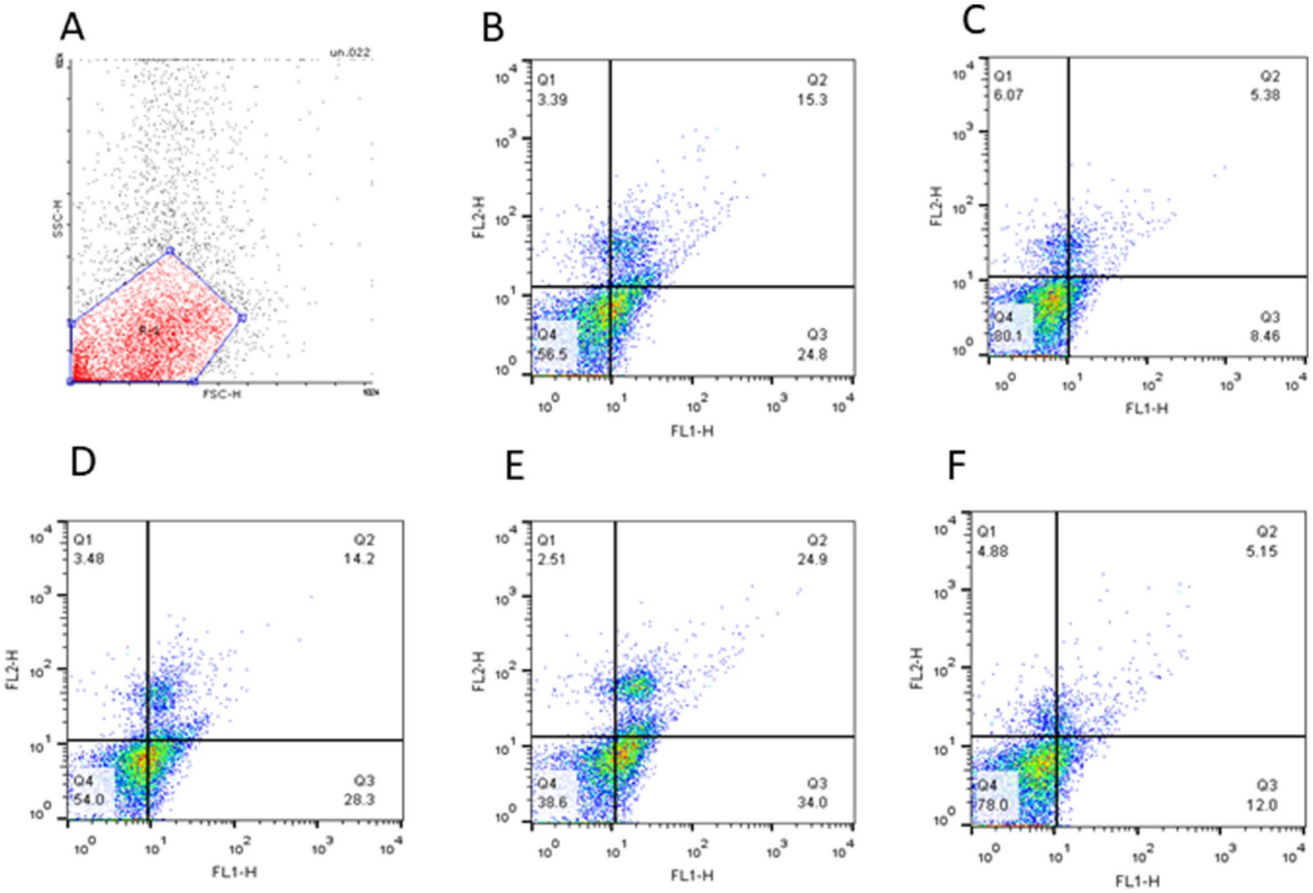
The quadrant plot of cells stained with Annexin-V and PI (FL1: Annexin-V fluorescence, FL2: PI fluorescence) showed Simmental bull spermatozoa treated with different concentrations of Y-27632 (**A**: negative control; **B**: control; **C**: 10 μM, **D**: 20 μM; **E**: 30 μM, and **F**: 40 μM Y-27632). The lower-left quadrant (Q4) represents spermatozoa negative for both Annexin-V and PI (A^−^/PI^−^; viable sperm). The lower-right quadrant (Q3) represents early apoptotic spermatozoa positive for Annexin-V but negative for PI (A^+^/PI^−^). The upper-right quadrant represents (Q2) late apoptotic cells positive for both Annexin-V and PI (A^+^/PI^+^). The upper-left quadrant (Q1) represents necrotic cells negative for Annexin-V but positive for PI (A^−^/PI^+^).

The changes associated with the apoptosis status of frozen–thawed spermatozoa treated with various concentrations of Y-27632 are shown in Table 6 and Figures 2A–E. Compared to other groups, the addition of 20 μM Y-27632 to the freezing extender significantly (*p* < 0.05) increased the percentage of viable cells according to the flow cytometric analysis of thawed spermatozoa. Different concentrations of Y-27632 did not affect the percentage of early apoptotic cells (*p* > 0.05). Compared to other treatment groups, sperm supplementation with 20 μM Y-27632 significantly reduced the percentage of primary apoptotic cells (*p* < 0.05). The percentage of late apoptotic cells was significantly decreased in cells treated with 20 μM Y-27632 compared to spermatozoa treated with 40 μM Y-27632. The addition of different concentrations of Y-27632 to the freezing extender caused a decrease in the percentage of necrotic cells compared to the control group; however, such a difference was not statistically significant.

**Table 6:** Flow cytometric analysis of apoptosis statues in frozen–thawed bull spermatozoa supplemented with various ROCK inhibitor (Y-27632) concentrations.

			Concentration of Y-27632			
Variable (unit)	0 μM (Control)	10 μM	20 μM	30 μM	40 μM	*p*-value
Viable cell (%)	55.36 ± 3.72^a^	58.55 ± 1.02^a^	74.26 ± 3.32^b^	53.70 ± 3.21^a^	53.26 ± 2.94^a^	*p* = 0.003
Early apoptotic cell (%)	19.57 ± 5.49^ab^	25.65 ± 1.00^b^	12.68 ± 2.12^a^	30.50 ± 4.44^b^	23.79 ± 1.11^b^	*p* = 0.034
Late apoptotic cell (%)	15.96 ± 1.29^ab^	10.84 ± 2.89^ab^	7.95 ± 1.80^a^	12.94 ± 3.16^ab^	20.23 ± 2.53^b^	*p* = 0.041
Necrotic cell (%)	9.08 ± 4.97	4.95 ± 1.26	5.06 ± 0.52	2.84 ± 1.24	2.71 ± 0.76	*p* = 0.092

Values are presented as the means ± standard error of the Mean (SEM). ^a,b,c^Different superscripts in the same row indicate differences between treatment groups (*p* < 0.05); each experiment was performed at least three times and underwent statistical analysis. –, no significant difference (*p* > 0.05).

### Gene expression

**Figure 3:**
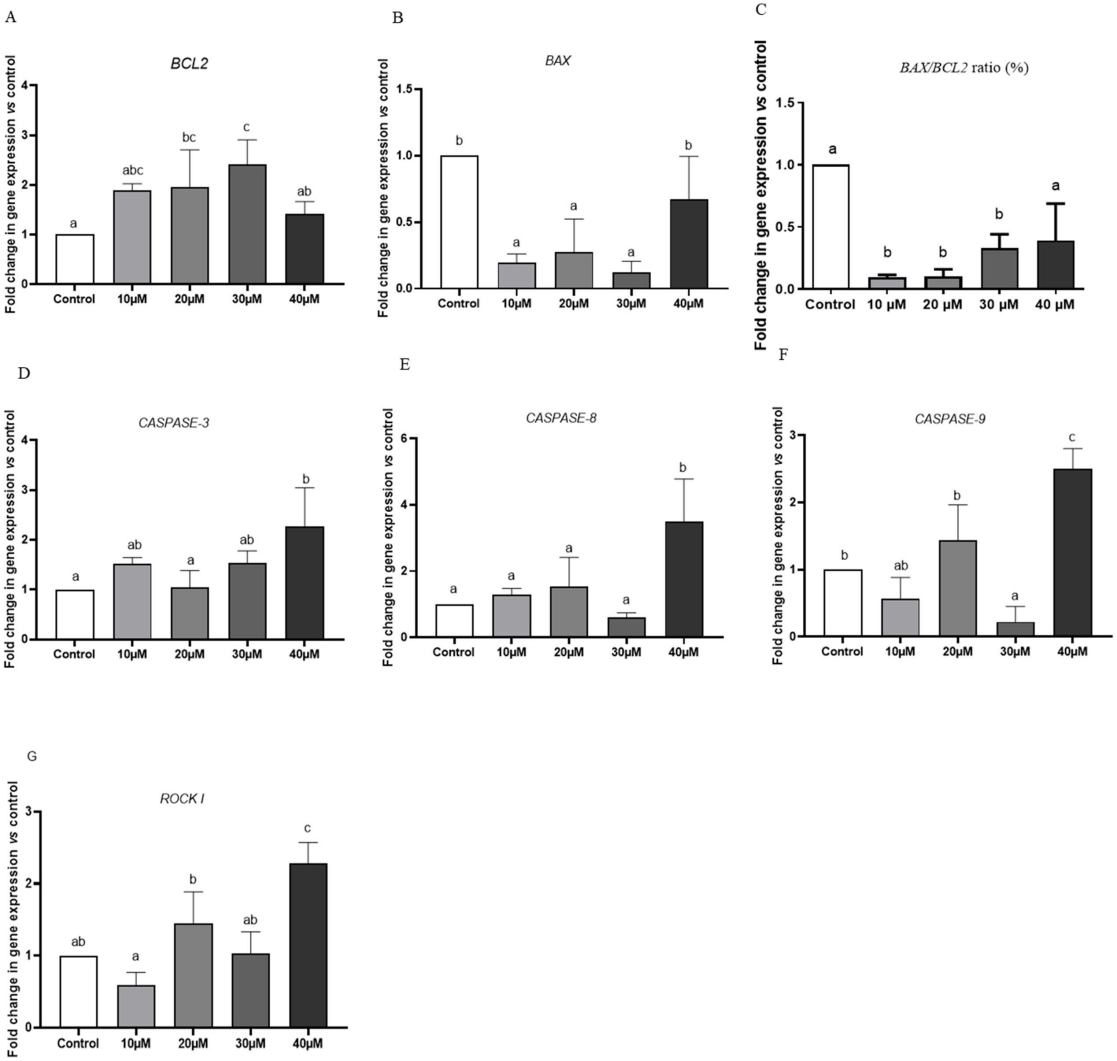
The effect of different concentrations of Y-27632 on the expression levels of *BCL2* **(A)**, *BAX* **(B)**, *BAX/BCL-2 RATIO* **(C)**, *CASPASE-3***(D)**, *CASPASE-8* **(E)**, *CASPASE-9* **(F)**, and *ROCK I* **(G)** genes in frozen–thawed spermatozoa. Target genes were normalized against the internal control gene (*GAPDH*), and results are expressed as fold changes relative to the control. Values are expressed as means ± SEM. ^a,b,c^Different superscript letters on the bar graph indicate significant differences (*p* < 0.05) compared with other treatment groups. The difference between the experimental groups was analyzed by one-way ANOVA followed by Tukey’s *post hoc* test. In contrast, statistically no significant differences have been indicated by the same letters.

Compared with the control group, the expression level of *BCL2* was approximately two-fold higher in spermatozoa treated with 20 μM Y-27632 (Figure 3A). However, no statistically significant (*p* > 0.05) difference was detected in the mRNA level of *BCL2* in spermatozoa treated with 10, 20, and 30 μM Y-27632 compared to the control group (Figure 3A). The addition of 10, 20, and 30 μM Y-27632 to the freezing extender caused a significant decrease in *BAX* gene expression in post-thaw spermatozoa compared to the control group and cells treated with 40 μM Y-27632 (Figure 3B). The ratio of *BAX/BCL2* expression was significantly decreased in spermatozoa treated with 10, 20, and 30 μM Y-27632 compared to the control group (*p* < 0.05; Figure 3C). Supplementation of the freezing extender with 20 μM Y-27632 significantly (*p* < 0.05) decreased the relative mRNA level of *CASPASE-3* compared to cells treated with 40 μM Y-27632 (Figure 3D). Moreover, no significant difference was observed in the expression levels of CASPASE-3 between spermatozoa treated with 10, 20, and 30 μM compared to the control group (*p* > 0.05; Figure 3D). The relative mRNA expression of the *CASPASE*-8 gene was significantly (*p* < 0.05) lower in spermatozoa treated with 10–30 μM Y-27632 compared to cells treated with 40 μM Y-27632 (Figure 3E). There were no significant differences in the expression of the *CASPASE*-8 gene between spermatozoa treated with 10, 20 μM Y-27632 and the control group (*p* > 0.05; Figure 3E). The relative expression of the *CASPASE-9* gene was significantly (*p* < 0.05) lower following the treatment of spermatozoa with 30 μM Y-27632 compared to those treated with 20 and 40 μM Y-27632 and the control group (Figure 3F). There were no significant differences in the expression rate of *CASPASE-9* between spermatozoa treated with 10 and 30 μM Y-27632 (*p* > 0.05; Figure 3F). The addition of 10 μM Y-27632 to the freezing extender significantly (*p* < 0.05) reduced the relative mRNA level of the *ROCK I* gene compared to spermatozoa treated with 20 and 40 μM Y-27632. There were no significant differences in the expression level of *ROCK I* between cells treated with 10 and 30 μM Y-27632 (*p* > 0.05; Figure 3G).

### *In vitro* embryo production (IVEP)

The results of IVEP showed that the cleavage rates were significantly higher after fertilization with post-thaw spermatozoa treated with 20 μM and 30 μM Y-27632 compared with the cells treated with 40 μM Y-27632 and the control group. The morula rates were significantly higher after fertilization with post-thaw spermatozoa treated with 10 and 20 μM Y-27632 compared with the control group (*p* < 0.05; Table 6). Fertilization of oocytes with post-thaw spermatozoa treated with 10 and 20 μM Y-27632 significantly increased (*p* < 0.05) the blastocyst rate compared to spermatozoa treated with 40 μM Y-27632 and the control group (Table 7).

**Table 7:** Mean percentage ± standard error (SEM) of fertilizing ability and subsequent embryo development rate following *in vitro* fertilization with frozen–thawed sperm treated with different concentration of ROCK inhibitor (Y-27632).

Variables (unit)	Oocyte	Cleavage rate (%) (days 3)	Morula rate (%) (day 5)	Blastocyst rate (%) (day 7–8)
0 μM (Control)	100	64.33 ± 2.44^ab^	27.37 ± 5.32^a^	22.30 ± 3.51^ab^
10 μM	95	68.38 ± 2.64^bc^	44.74 ± 3.94^c^	30.72 ± 1.31^c^
20 μM	100	74.17 ± 3.63^c^	44.58 ± 2.79^c^	33.41 ± 2.88^c^
30 μM	102	74.30 ± 3.47^c^	40.58 ± 0.67^bc^	28.20 ± 3.15^bc^
40 μM	105	59.75 ± 2.47^a^	33.48 ± 3.56^ab^	20.77 ± 1.72^a^
*p*-value		0.0075	0.0075	0.0073

Values are presented as the means ± standard error of the mean (SEM). Within the columns, the different superscript letters (^a,b,c^) denote values that differ significantly (*p* < 0.05). –, no significant difference (*p* > 0.05). Each experiment was performed at least three times and underwent statistical analysis.

## Discussion

The present study examined how Y-27632 concentrations affect frozen–thawed sperm quality and embryonic development after IVF. The most significant parameters damaged during sperm freezing include the cytoskeleton and genomic structures, sperm kinematic parameters, sperm membrane integrity, mitochondrial membrane potential, and DNA fragmentation (45, 46). In addition, the expression of apoptosis-related genes that were activated during cryopreservation and could be inhibited by ROCK inhibitor was the primary emphasis of this study.

Our findings indicated that the highest frozen–thawed sperm motility was recorded with the extender containing 20 μM of Y-27632. This result is in agreement with the findings of Tharasanit et al. (27), who demonstrated that the addition of 10 μM ROCK inhibitor to feline semen extender improved sperm motility. Similarly, Karaşör et al. (28) indicated that the supplementation of the extender containing buck’s semen with 5 or 10 μM Y-27632 enhanced post-thaw sperm motility. Semen density, quantity, cryoprotectant doses, sensitivity to freezing, and plasma membrane variations across species may contribute to the different doses of Y-27632 required to achieve the same effect. It is conceivable that proliferative antioxidant additives act as a coenzyme in mitochondrial oxidative decarboxylation metabolism, which may explain why ROCK inhibitors appear to enhance the motility properties of bull semen. ROCK inhibitors aid in the reduction of mitochondrial dysfunction in sperm metabolism, which is required for ATP production and sperm motility (47, 48). In addition, ROCK inhibitors are able to increase sperm motility by affecting the cytoskeleton and microtubules as well as boosting sperm resistance against ROS (47). The present study showed that high doses of ROCK inhibitor (Y-27632), when added to the freezing extender, caused a decrease in sperm motility after thawing. Likewise, Zhang et al. (49) found that ROCK inhibitors had deleterious effects on boar oocyte maturation and actin-mediated somatic cell cytokinesis at high concentrations. Due to increased ROS production, high doses of ROCK inhibitors may reduce sperm motility. Increased generation of ROS disrupts mitochondrial function, promotes intracellular enzyme efflux, and impairs several sperm axonemal proteins, resulting in reduced sperm motility (50, 51).

The results of our study indicated that low doses of Y-27632 can prevent membrane lipid peroxidation and plasma membrane conformational changes during freezing by reducing ROS production. In this study, we observed the antioxidant effect of Y-27632 during the cryopreservation of bull sperm. The results indicated that supplementation of the freezing extender with 20 μM Y-27632 led to a decrease in lipid peroxidation. This property may be related to an increase in the antioxidant capacity of the compound against oxidants (52). In this study, the highest quality sperm in terms of membrane function and acrosome integrity were obtained when sperm samples were frozen with 20 μM Y-27632. Our findings were in agreement with the results of Watanabe et al. (24), who studied the impact of low doses (10 μM) Y-27632 on the plasma membrane integrity of spermatozoa. They showed that ROCK inhibitors reduced subtle alterations rather than completely protecting spermatozoa from physical damage during cryopreservation. The antioxidant activity of ROCK inhibitors may be related to improving sperm plasma membrane integrity by reducing plasma membrane fluidity, thus protecting sperm from lipid peroxidation. In addition, Y-27632 was shown to promote Akt activation in cells, perhaps by controlling PTEN activation (53). The activation of Akt maintains the integrity of the plasma membrane of spermatozoa by inhibiting caspase-3 and -7, leading to a decrease in apoptosis in these cells (54). In contrast to our findings, Karaşör et al. (28) indicated that the addition of 5 or 10 μM Y-27632 to the freezing extender containing buck semen did not improve plasma membrane and acrosome integrity. This inconsistency may be due to variations in the composition of the sperm plasma membrane and seminal plasma between the two species (28, 55).

In the present study, the highest percentage of normal morphology was obtained when sperm samples were cryopreserved with 10 μM of Y27632. However, the lowest percentage of normal morphology was detected in sperm specimens treated with 40 μM of Y27632. Previous studies have shown that a difference in sperm morphology depends on the status of the sperm membrane and acrosome (56). In addition, Said et al. (57) found that increased ROS production leads to an increased number of spermatozoa with mid-piece defects, such as the presence of residual extracellular cytoplasm. It can be concluded that the high concentration of Y-27632 cannot prevent the negative effects of the freeze–thaw process, and since many spermatozoa suffer from morphological damage, the functional integrity of the acrosome and plasma membrane is compromised.

Apoptosis is a type of programmed cell death in which genetically defective cells are removed (58). The biochemical characteristics of apoptosis are the transport of phosphatidylserine to the plasma membrane, activation of caspases, and DNA fragmentation (59). Our finding indicated that the percentage of dead cells and those with apoptotic-like changes in spermatozoa treated with 20 μM Y-27632 was considerably lower than in the control group. Our findings were consistent with previous research demonstrating that Y-27632 can effectively inhibit apoptosis and increase the viability of spermatozoa following the freeze–thaw process, as well as other cell types such as hESCs (24). The increase in the number of post-thaw viable spermatozoa at low doses of Y-27632 may be attributable to an improvement in plasma and acrosome membrane integrity and a decrease in the production of ROS, leading to a reduction in apoptosis via the inactivation of the internal and external pathways of apoptosis (60).

The present study demonstrated that the supplementation of the freezing extender with 30 μM Y-27632 decreased the expression of *B*AX while reducing the expression of *BCL2*. *BAX* is a known pro-apoptotic protein released in response to stressors in male germ cells and acts through the intrinsic signaling pathway (61). *BAX* expression levels determine cell survival or death, and the *BCL2/BAX* ratio is considered an indicator of apoptosis status. Caspases are a family of proteases essential for the regulation of apoptosis (62). Studies have shown that cryopreservation activates *caspase-3*,*-8*, and *-9* through intrinsic and extrinsic apoptotic pathways (17). The role of caspases in death receptor signaling has been demonstrated by biochemical and molecular studies (63). In this study, the mRNA level of *CASPASE*-3 was significantly decreased following the supplementation of the freezing extender with 20 μM Y-27632. Also, the expression levels of *caspase-8* and -9 were diminished after the treatment of spermatozoa with 30 μM Y-27632. The reduced expression levels of BAX and *CASPASE-3,-8*, and *-9* genes, as well as the increased expression of *Bcl-2* in frozen–thawed bull spermatozoa with lower doses of Y-27632, supported the fact that Y-27632 acts as an apoptosis inhibitor.

The results of the current study indicated that the treatment of the freezing extender containing bull semen with a low dose of Y-27632 could also positively affect *in vitro* embryonic development. In the current research, spermatozoa treated with 10 and 20 μM Y-27632 developed a higher percentage of blastocysts than spermatozoa treated with 40 μM Y-27632. The greater percentage of blastocyst development rate might have been due to better quality of spermatozoa in response to a low dose of Y-27632. A significant body of research has demonstrated that Y-27632 is a potent and selective Rho kinase inhibitor that increases the survival of mammalian embryos after the freeze–thaw process (23, 64). Y-27632 inhibits the RhoA/ROCK pathway, which is involved in cell growth and apoptosis (24). ROCK inhibitor (Y-27632) has been successfully used to reduce programmed cell death of embryonic stem cells (ESCs) and oocytes (65, 66).

Finally, it seems supplementation of freezing extenders with low concentration of Y-27632 by decreasing intracellular ROS levels causes improved motility, plasma membrane stability, acrosome integrity, as well as DNA integrity. Similar to the study conducted on human spermatozoa, these results showed that inhibiting the Rock signaling pathway can protect sperm from cold shock (53). On the other hand, during the freeze–thaw procedure, activation of caspase enzymes leads to the initiation of apoptosis and consequent fragmentation of sperm chromatin (67). A growing body of literature, the significant association between DNA integrity and the fertilizing potential of spermatozoa was confirmed (68). The integrity of sperm chromatin affects not only fertilization success but also embryo development and the live birth rate (69). As a result, the higher fertilization and blastocyst rates can be related to the increased DNA integrity in spermatozoa treated with low doses of Y-27632.

## Conclusion

It can be concluded that the effects of Y-27632 on sperm parameters are dose-dependent. Supporting the idea that Y-27632 has anti-apoptotic and antioxidant effects in the bull freezing extender, studies at intermediate doses (20 μM) showed a reduction in apoptosis-related gene expression, as well as in ROS production and MDA levels. Furthermore, this concentration improves the quality of sperm kinematics and the functional parameters and decreases DNA fragmentation, leading to an increased blastocyst rate. The precise mechanism of the ROCK inhibitors in controlling apoptosis during cryopreservation can be better understood in future studies by analyzing the proteins involved in the Rho/Rock pathway. Furthermore, it would be interesting to determine if supplementing freeze extenders with ROCK inhibitors improves pregnancy rates following artificial insemination in different species.

## Data availability statement

The raw data supporting the conclusions of this article will be made available by the authors, without undue reservation.

## Ethics statement

The animal study was reviewed and approved by the Animal Ethics Board of the University of Tabriz (IR.TABRIZU.REC.1401.045). Written informed consent was obtained from the owners for the participation of their animals in this study.

## Author contributions

MB: semen samples collection, designing methodology, and writing the manuscript. RA: direction and supervision of the study, data analysis, and drafting the manuscript. TT: study design, and reviewing and editing of the drafted manuscript. HH: reviewing and editing of the drafted manuscript. All authors contributed to the article and approved the submitted version.

## Conflict of interest

The authors declare that the research was conducted in the absence of any commercial or financial relationships that could be construed as a potential conflict of interest.

## Publisher’s note

All claims expressed in this article are solely those of the authors and do not necessarily represent those of their affiliated organizations, or those of the publisher, the editors and the reviewers. Any product that may be evaluated in this article, or claim that may be made by its manufacturer, is not guaranteed or endorsed by the publisher.
